# Early cardiac-chamber-specific fingerprints in heart failure with preserved ejection fraction detected by FTIR and Raman spectroscopic techniques

**DOI:** 10.1038/s41598-022-07390-2

**Published:** 2022-03-02

**Authors:** Niki Tombolesi, Raffaele Altara, Gustavo J. J. da Silva, Cynthia Tannous, Fouad A. Zouein, Kåre-Olav Stensløkken, Assunta Morresi, Marco Paolantoni, George W. Booz, Alessandro Cataliotti, Paola Sassi

**Affiliations:** 1 Department of Chemistry, Biology and Biotechnology, University of Perugia, Via Elce di Sotto 8, 06123 Perugia, Italy; 2 Institute for Experimental Medical Research (IEMR), Oslo University Hospital and University of Oslo, Ullevål, Kirkeveien 166, Build.7, 0450 Oslo, Norway; 3 KG Jebsen Centre for Cardiac Research, University of Oslo, Oslo, Norway; 4 Department of Pathology, School of Medicine, University of Mississippi Medical Center, Jackson, MS USA; 5 Department of Pharmacology and Toxicology, Faculty of Medicine, American University of Beirut Medical Center, Riad El-Solh, Beirut, Lebanon; 6 The Cardiovascular, Renal, and Metabolic Diseases Center of Excellence, American University of Beirut Medical Center, Riad El-Solh, Beirut, Lebanon; 7 Department of Pharmacology and Toxicology, School of Medicine, University of Mississippi Medical Center, Jackson, MS USA; 8 Department of Molecular Medicine, Institute for Basic Medical Sciences, University of Oslo, Oslo, Norway

**Keywords:** Cardiovascular biology, Heart failure

## Abstract

The pathophysiology of heart failure with preserved ejection fraction (HFpEF) is a matter of investigation and its diagnosis remains challenging. Although the mechanisms that are responsible for the development of HFpEF are not fully understood, it is well known that nearly 80% of patients with HFpEF have concomitant hypertension. We investigated whether early biochemical alterations were detectable during HFpEF progression in salt-induced hypertensive rats, using Fourier-transformed infrared (FTIR) and Raman spectroscopic techniques as a new diagnostic approach. Greater protein content and, specifically, greater collagen deposition were observed in the left atrium and right ventricle of hypertensive rats, together with altered metabolism of myocytes. Additionally, Raman spectra indicated a conformational change, or different degree of phosphorylation/methylation, in tyrosine-rich proteins. A correlation was found between tyrosine content and cardiac fibrosis of both right and left ventricles. Microcalcifications were detected in the left and right atria of control animals, with a progressive augmentation from six to 22 weeks. A further increase occurred in the left ventricle and right atrium of 22-week salt-fed animals, and a positive correlation was shown between the mineral deposits and the cardiac size of the left ventricle. Overall, FTIR and Raman techniques proved to be sensitive to early biochemical changes in HFpEF and preceded clinical humoral and imaging markers.

## Introduction

Heart failure (HF) is a major clinical challenge that is associated with a markedly high risk of death^1^. It affects over 26 million people worldwide, accounts for the majority of hospitalizations among the elderly, and its prevalence continues to rise. Approximately half the patients with HF have normal contractility. This medical condition of HF with preserved ejection fraction (HFpEF) is characterized by cardiac hypertrophy and increased stiffness of the heart, which compromise ventricular relaxation and reduce stroke volume^2,3^. Metabolic diseases such as obesity, diabetes and hypertension often contribute to the development of HFpEF^4^. Diagnosis of HFpEF is a challenge that relies upon the presence of symptoms and/or signs of HF, preserved left-ventricular systolic function, and evidence of diastolic dysfunction. The diagnosis of HFpEF is often belated and the treatment of this condition remains largely unsuccessful, with a five-year survival rate of 43% after a first diagnosis^5^.

The underlying etiopathogenetic mechanisms that lead to overt HFpEF remain unclear. While most research has focused on the remodeling of the left ventricle (LV) in HFpEF, there are compelling reasons to think that events in the left atrium (LA) and right ventricle (RV) contribute in a major manner to HFpEF^6,7^. Indeed, LA dysfunction may explain the pulmonary congestion, shortness of breath, and exercise intolerance that are associated with HFpEF. Accumulating evidence indicates that right HF accounts for more than 50% of deaths in patients with HFpEF^8^. However, these events are not well studied and remain poorly understood. Evidence indicates that biochemical changes in the cells and tissues precede alteration of function and structure of the organs, and it is plausible that rapid detection of such chemical changes could lead to early diagnosis of the disease and uncover yet unknown pathogenetic mechanisms of dysfunction.

In this study, we aimed to explore the use of Fourier-transform infrared (FTIR) and Raman spectroscopies to address this knowledge gap and to provide a biochemical profile of the remodeling events that occur in all four chambers of the heart during the evolution of HFpEF. The diagnostic utility of FTIR and Raman spectroscopy in the HFpEF setting have not been investigated before; hence, we are pioneering the use of these technologies in the cardiovascular arena, and intend to expand their use to clinical practice for early diagnosis of cardiac diseases. We first hypothesized that the combined use of micro-FTIR and Raman spectroscopy would enable detection of novel chemical fingerprints and structural modifications that are correlated with the cardiac alterations that are known to lead to HFpEF. We further hypothesized that, during evolution toward HFpEF, due to the different stresses that occur in the four cardiac chambers, the biochemical modifications of the atria would be different from those of the ventricles. To test these hypotheses, we investigated the progression of diastolic dysfunction and cardiac hypertrophy through application of the spectroscopic techniques to a rat model of salt-induced hypertension.

## Materials and method

All experiments were performed in accordance with relevant guidelines and regulations.

### Experimental group and study protocol

The following description follows the recommendations of the ARRIVE guidelines. The experimental protocol was approved by the Committee for Animal Research, Forsøksdyrforvaltningens tilsyns- og søknadssystem protocol number 12582, which is under the auspices of the Norwegian Food Safety Authority (Mattilsynet). Twenty Dahl/salt-sensitive (Dahl/SS) male rats with an approximate initial weight of 150 g were used. The rats were purchased from Charles River Laboratories (Wilmington, MA, USA) and housed in a room with a 12-h/12-h light/dark cycle at a temperature of 21 °C and a humidity level of 55%. They were maintained on a normal salt (NS) diet until they were seven weeks old. Normal salt (0.3% NaCl) diet was from Special Diets Services, United Kingdom. A total of 11 Dahl/SS rats were then randomly switched to a high-salt (HS) diet, which contained 4% NaCl, to induce high blood pressure^9^. Rats on the HS diet were labeled as hypertensive (HT). Nine rats were kept normotensive (NT) on the NS diet. Drinking water and food were provided ad libitum. Rats were sacrificed six weeks (NT = 3, HT = 3), 16 weeks (NT = 3, HT = 4), and 22 weeks (NT = 3, HT = 4) after initiation of the diet regime.

Animals were sacrificed via a euthanasia method involving deep anesthesia (5% isoflurane), exsanguination and ultimately heart excision, consistent with the American Veterinary Medical Association (AVMA) Guidelines for the Euthanasia of Animals (2020). Heart tissue was collected at sacrifice and immediately processed. Atria were separated from the ventricles and snap-frozen separately in liquid nitrogen. Ventricles were sectioned at the mid-transverse plane. The portions from the midpoint to base of the heart were separated according to whether they contained the LV or RV, and snap-frozen. Six, 16 and 22 weeks into the HS diet, levels of N-terminal pro-brain natriuretic peptide (NT-proBNP) in plasma were measured by use of a commercially available kit (Cusabio Technology, Wuhan, China).

### Echocardiography

Cardiac function was assessed by application of transthoracic echocardiography (ECG) through use of a Vevo 3100 high resolution in-vivo imaging system from VisualSonics (Toronto, Canada). Echocardiographic assessments were made after 6, 16, and 22 weeks of the HS diet. Briefly, animals were maintained under anesthesia (1.5–2% isoflurane mixed with oxygen) on a pre-warmed ECG transducer pad while body temperature and ECG were monitored. Measurements were made with either MX250 or MX550D transducers, frequency set at 20–25 MHz. M-mode measurements in the parasternal, long-axis view were obtained to assess the functions and dimensions of the LVs and LAs. The left ventricular ejection fraction (LVEF) was calculated as:

$$ {1}00 \, * \, \left( {\left( {{\text{LV Vol}};_{{\text{d}}} {-}{\text{ LV Vol}};_{{\text{s}}} } \right)/{\text{LV Vol}};_{{\text{d}}} } \right), $$

in which LV Vol;_d_ is the left ventricular volume at end diastole and LV Vol;_s_ is the left ventricular volume at end systole.

The mass of the LV was estimated by the formula:

$$ {1}.0{53 }* \, \left( {\left( {{\text{LVID}};_{{\text{d}}} + {\text{ LVPW}};_{{\text{d}}} + {\text{ IVS}};_{{\text{d}}} } \right)^{{3}} {-}{\text{ LVID}};_{{\text{d}}}^{{3}} } \right), $$

in which LVID;_d_ represents the left ventricular internal diameter at end diastole, LVPW;_d_ represents the thickness of the left ventricular posterior wall at end diastole and IVS;_d_ is the interventricular septum thickness at end diastole.

Relative wall thickness (RWT) was calculated as:

$$ {2 }*{\text{ LVPW}};_{{\text{d}}} /{\text{ LVID}};_{{\text{d}}} \left[ {{17}} \right]. $$

The strength of E and A waves in left ventricular filling velocities were assessed via pulsed-wave Doppler in the parasternal, long-axis view. ECG analyses were performed by an operator who was blinded to the identity of the groups.

### Blood pressure measurement

Blood pressure was measured through use of the CODA non-invasive blood-pressure acquisition system for rats (Kent Scientific Corporation, Torrington, CT, USA). Animals were kept in restraint tubes that were placed over a heating platform (preheated to 33–35 °C) and blood pressure was measured by a tail-cuff system (cuff size, large; rat holder, large rat 300–500 g). Each recording session consisted of 25 acclimatization cycles (not used in the analysis), followed by 20 inflation and deflation cycles (the occlusion cuff was inflated to 250 mmHg and deflated over 20 s). Rats were trained for at least five consecutive days before blood pressure measurements were recorded. Measurements were recorded after six, 16, and 22 weeks of the HS diet.

### Cardiac collagen content assessment by histochemistry

Hearts were excised, rinsed in phosphate-buffered saline (PBS), quickly blotted on gauze and then fixed in 10% formalin for a minimum of 24 h. The bi-ventricular apex of each heart and atrium were embedded in paraffin and cut into 4 µm sections. Sections were stained with Masson’s trichrome (Polysciences Inc., Warrington, Pennsylvania, USA) to assess collagen abundance. Stained sections were scanned (20× magnification) with an AxioScan Z1 instrument (Zeiss, Jena, Germany), and interstitial and perivascular fibrosis areas (%) were quantified using ZEN2 blue edition software (Zeiss, Jena, Germany). All histological quantifications were performed independently by an experienced researcher who was blinded to group identity.

### FTIR and Raman measurements

FTIR spectra were collected in transmission mode from 900 to 3800 cm^−1^ at 2 cm^−1^ resolution by using a Bruker Tensor 27 spectrometer and a Hyperion 3000 microscope that was equipped with a 15 × Cassegrain objective and a 64 × 64 pixel focal-plane-array detector. Areas that measured 180 × 180 µm (2.8 µm pixel resolution) were analyzed by averaging 256 measurements.

The confocal Raman microscope (Olympus IX73 inverted microscope coupled to the S&I MonoVista CRS + spectrometer) was used in backscattering mode by exciting the sample at 785 nm through a 50× objective lens (numerical aperture (NA) = 0.50), and collecting the 200–1800 cm^−1^ spectral range with a spectral resolution of 3 cm^−1^.

Spectra of snap-frozen and formalin-fixed, paraffin-embedded samples were compared as reported in the Supplementary Material (SM) section, to investigate how much the results are influenced by the sample treatment prior to spectra acquisition. In view of these observations, to inspect the biochemistry of heart tissues, we analyzed snap-frozen samples from the four cardiac chambers of both normal and HFpEF-prone rats. Interpretation of all spectra was done by visual inspection.

### Statistical analysis

To compare different samples, 64 × 64-pixel FTIR images (see Fig. 2SM) were collected in three different regions of each section. These regions were randomly selected over the entire sample area in order to obtain a spectrum which is representative of the sample as a whole and not of a particular region. More than 100 spectra were selected from each image and normalized to the 2850 cm^−1^ intensity. A quality test was performed on IR data based on the intensity of the amide I peak at 1650 cm^−1^ ca., and the noise in the region between 1800 and 1900 cm^−1^, we rejected spectra having this ratio lower than 80. The normalized second derivatives of the resulting spectra were averaged and standard deviations were evaluated with Opus 8.1 software from Bruker Optics (Billerica, MA, USA). For each sample category (related to the type of heart chamber and to the type of rat, normotensive (NT) or hypertensive (HT)), we averaged data from three different replicas. Raman spectra were obtained from 10 different points of each tissue section (see Fig. 3SM). A baseline correction was performed on the spectra subtracting a polynomial curve passing through the points at 500, 800, 1130, 1500 and 1750 cm^−1^. Baseline correction, average profiles and standard deviations were obtained with Opus 8.1 software from Bruker Optics. To obtain a representative spectral profile of the four heart chambers of both NT and HT samples, we further averaged the data of the same sample category (three replicas). To evaluate the intensity ratio of Raman spectral components at 830–860 cm^−1^ and at 940–960 cm^−1^, the integrated intensities of bands with their standard uncertainties were determined by performance of the integration routine of the Opus 8.1 software. The relative uncertainties of the intensity ratios were evaluated from the sum of the relative uncertainties of integrated intensities.

Ordinary one-way analysis of variance (ANOVA) was used to test the progressive change of levels of the circulating NT-proBNP at 16 and 22 weeks within each group. A t-test was used to determine the difference between the levels in NT and HT rats at each time period. The Pearson correlation method was used to assess the strength of the association between cardiac phenotype and the FTIR/Raman findings. *P* values < 0.05 were considered statistically significant. Values are reported as mean ± standard error of measurement (SEM). Statistical tests were performed with GraphPad Prism 8.0.1 (San Diego, CA, USA).

### Ethics approval

The experimental protocol was approved by the Committee for animal research, which is under the auspices of the Norwegian Food Safety Authority (Mattilsynet) (FOTS protocol number 12582).

### Consent for publication

All authors have given their consent for publication.

## Results

### FTIR and Raman assessments of the four heart chambers in normotensive rats

**Figure 1:**
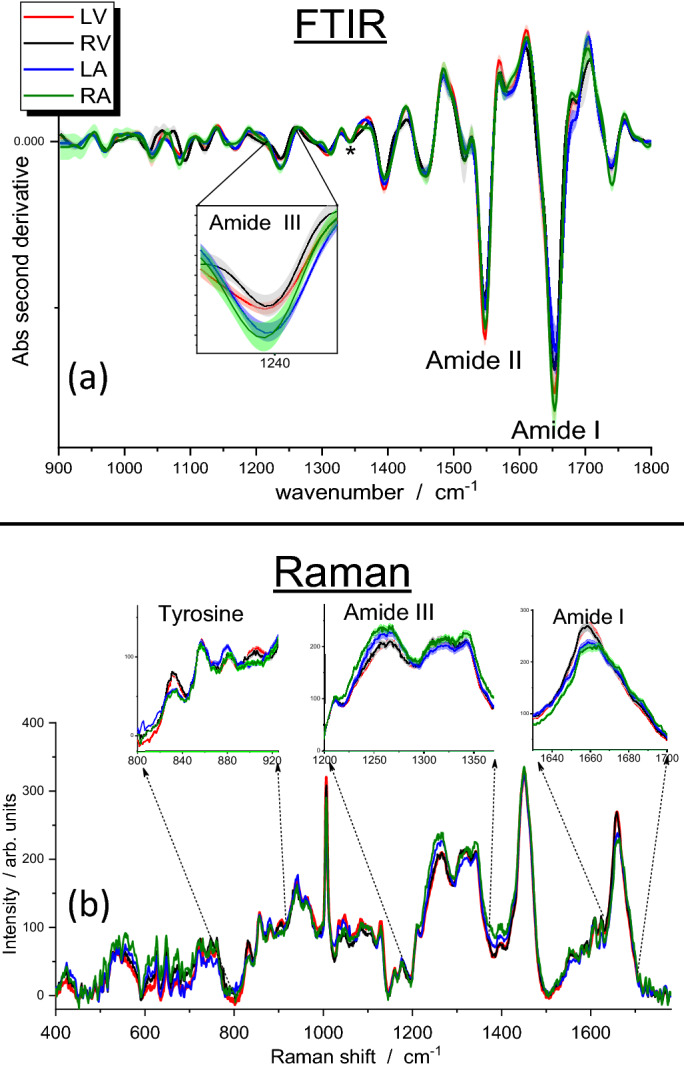
(**a**) Second-derivative FTIR spectra of the four heart chambers of normotensive Dahl/SS rats. The standard deviations of the different experimental profiles are represented by the shadowed areas. A magnification of the amide III region at about 1240 cm^−1^ is visualized in the inset. The asterisk marks the collagen band at approximately 1340 cm^−1^^35^; we did not observe significant differences among the spectra of the four heart chambers. (**b**) Raman spectra of the four heart chambers of normotensive rats. The standard deviations of the different experimental profiles are represented by the shadowed areas. A magnification of the amide I, amide III and 800–900 cm^−1^ regions are visualized in the insets to evidence the differences between spectra from ventricles and atria. LV indicates left ventricle; RV, right ventricle; LA, left atrium; and RA, right atrium.

The main differences among the four heart chambers were represented by the amidic bands of proteins^10^ at approximately 1650 cm^−1^ (amide I), 1540 cm^−1^ (amide II), and 1250 cm^−1^ (amide III). They are described by the second-derivative FTIR spectra (Fig. 1). The amide III band showed an intense minimum at 1237 cm^−1^; at these wavenumbers, the major contribution came from collagen^11^. The highest intensity of these amidic bands was shown in the spectrum of the RA; in the case of the amide III band, a net separation enabled atria to be distinguished from ventricles.

All Raman spectra were normalized to the 1450 cm^−1^ band, which is mainly related to the lipid concentration in the tissue (Fig. 1b). The amide III band, at about 1250 cm^−1^, showed a higher intensity in the atria compared with the ventricles. This finding corroborated our results from the FTIR analysis. Moreover, the amide I band on the high-frequency side of the investigated range showed a blue-shift and a lower intensity in the LA and RA than in the LV and RV. The Raman analysis also demonstrated a higher intensity of spectral components at 830 and 903 cm^−1^ in LV and RV samples than in the LA and RA.

### Time-course assessment of adverse cardiac remodeling in Dahl/SS rats

**Figure 2:**
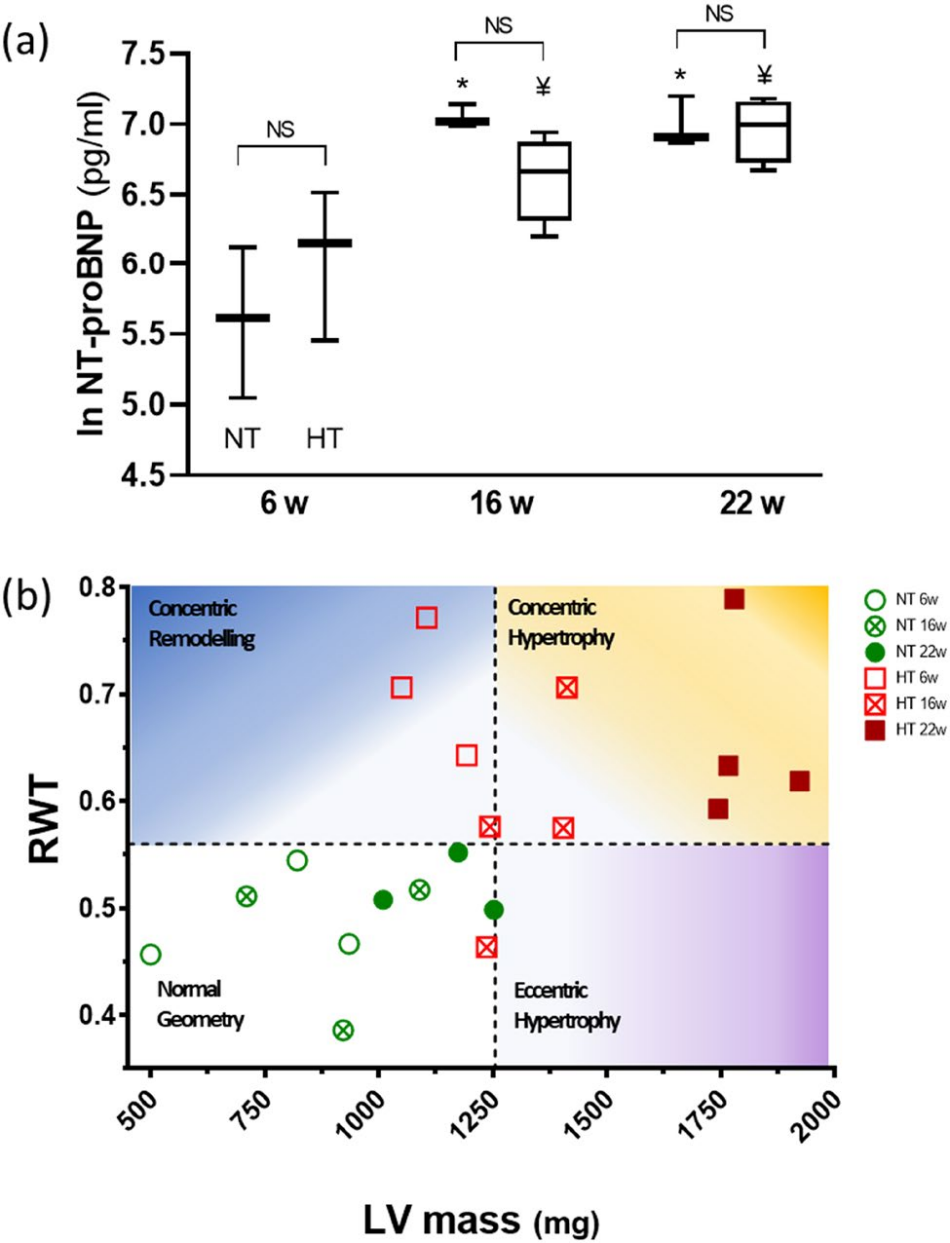
(**a**) Plasma NT-proBNP at 16 and 22 weeks increased in both normotensive (NT) and salt-fed (HT) Dahl/SS rats compared with baseline. *P ≤ 0.05 and ¥ ≤ 0.05 versus same group at 6 weeks. Data are paired as NT and HT (box and Tukey whiskers). (**b**) The relationship between LV mass and RWT was used to evaluate the type of remodeling that occurred in the Dahl/SS rats that were fed either NS or HS food. All rats that were fed a NS diet displayed a normal geometry (white, bottom-left quadrant). Rats that were fed a HS diet for six weeks had an increased RWT, indicating a concentric remodeling compared with NT rats. At 16 weeks of a HS diet, Dahl/SS rats showed signs of concentric hypertrophy, which was ultimately fully developed after 22 weeks of HS diet. LV mass indicates left ventricular mass; RWT, relative wall thickness; NT, normotensive Dahl/SS rat; and HT, hypertensive Dahl/SS rat.

HS diet Dahl/SS rats progressively developed high blood pressure; there was an approximate increase of 20 mmHg after six weeks of the diet (HS6, *p* = 0.19), 29 mmHg at 16 weeks (HS16, *p* = 0.06), and 32 mmHg at 22 weeks (HS22, *p* = 0.02) in systolic blood pressure compared with their relative NT controls (Table 1). HS6 Dahl/SS rats showed initial signs of adverse cardiac remodeling: increased thickness of the walls, lengthened LA diameter, and concentric remodeling (Table 1). A prolonged 16-week diet exacerbated these signs of a cardiac condition, and deteriorated heart relaxation, as indicated by a decrease in E/A ratio (Table 1). Levels of NT-proBNP in plasma, which is a clinical measure of the degree of HF, progressively increased in HT rats from six to 22 weeks (*p* = 0.02). However, this increase was age related and was no different to that observed in the age-matched NT controls (Fig. 2a). HT rats transitioned from concentric remodeling into concentric hypertrophy, which was shown by an increase of LV mass in parallel with that of the relative wall thickness (Fig. 2b). HS22 Dahl/SS rats showed elevations in their mean arterial blood pressure, heart-weight to body-weight ratio, LV mass, thickness of the heart, and size of the LA, with a decreased E/A ratio (Table 1).

**Table 1:** Time-course hemodynamic, autoptic and echocardiographic main characteristics of normal and high-salt fed Dahl/SS rats.

	6w diet		16w diet		22w diet	
	NT (n = 3)	HT (n = 3)	NT (n = 3)	HT (n = 4)	NT (n = 3)	HT (n = 4)
SBP (mmHg)	161.0 ± 2.4	183.8 ± 2.4*	147.2 ± 4.9	176.9 ± 16.5	149.4 ± 3.5	181.1 ± 10.1*
DBP (mmHg)	112.4 ± 5.0	124.2 ± 11.8	98.0 ± 13.2	134.9 ± 24.8	106.4 ± 3.2	134.8 ± 12.3
MAP (mmHg)	128.3 ± 4.2	143.7 ± 10.2	114.0 ± 9.1	148.7 ± 21.7	120.5 ± 1.9	149.9 ± 11.4
BW (g)	331.7 ± 9.5	316.7 ± 4.6	425.0 ± 15.4	414.3 ± 10.6	420.7 ± 9.2	430.3 ± 12.3
HW (mg)	1212 ± 93.1	1342 ± 84.0	1284 ± 37.5	1455 ± 15.7*	1408 ± 29.9	1724 ± 59.6*
HW/BW	3.65 ± 0.1	4.23 ± 0.2	3.76 ± 0.1	4.62 ± 0.2	3.35 ± 0.1	4.0 ± 0.2
EF (%)	73.8 ± 1.5	81.5 ± 3.7	69.0 ± 2.1	70.2 ± 3.5	71.6 ± 1.6	66.1 ± 4.4
FS (%)	44.1 ± 1.3	51.7 ± 3.8	39.1 ± 0.5	41.5 ± 3.0	42.4 ± 1.4	38.3 ± 3.6
E/A	1.46 ± 0.04	1.08 ± 0.19	1.81 ± 0.09	0.98 ± 0.07*	1.82 ± 0.29	1.00 ± 0.17
LA (mm)	3.81 ± 0.09	5.01 ± 0.12*	3.62 ± 0.16	4.61 ± 0.32	3.96 ± 0.25	5.2 ± 0.15*
LV mass (mg)	883.1 ± 30.0	1001 ± 96.4	1189 ± 20.3	1324 ± 48.7	1145 ± 71.5	1803 ± 40.5*
LV Vol;_d_ (µl)	273.3 ± 6.2	220.4 ± 29.6	384.5 ± 21.9	345.0 ± 19.5	346.5 ± 34.1	394.1 ± 33.2
LV Vol;_s_ (µl)	71.4 ± 3.5	43.0 ± 14.6	118.2 ± 2.9	101.2 ± 8.2	98.5 ± 12.4	133.8 ± 19.8
IVS;_d_ (mm)	1.71 ± 0.08	1.87 ± 0.07	1.6 ± 0.05	1.8 ± 0.10	1.6 ± 0.26	2.0 ± 0.14
IVS;_s_ (mm)	2.86 ± 3.14	3.2 ± 0.13	2.6 ± 0.06	3.2 ± 0.14*	2.9 ± 0.13	3.5 ± 0.14*
LVPW;_d_ (mm)	1.75 ± 0.02	3.0 ± 0.06*	2.0 ± 0.14	2.3 ± 0.14	2.1 ± 0.03	2.8 ± 0.08*
LVPW;_s_ (mm)	2.77 ± 0.13	3.49 ± 0.05*	3.13 ± 0.17	3.38 ± 0.01	3.3 ± 0.02	3.9 ± 0.10*

### Early diagnosis of HFpEF by FTIR and Raman spectroscopy

**Figure 3:**
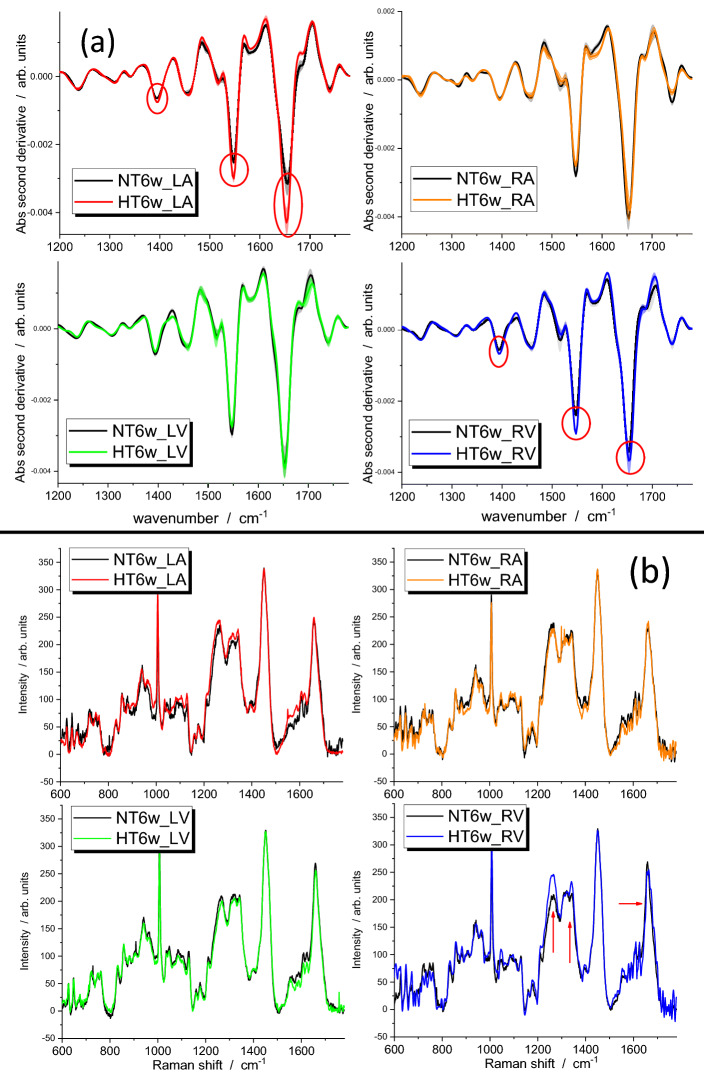
(**a**) Second-derivative FTIR spectra of the four heart chambers of normotensive and hypertensive (six-week, 6w) rats. Significant differences due to the pathological condition are shown by the red circles. (**b**) Raman spectra of the four heart chambers of normotensive and hypertensive (6w) rats. Red arrows indicate significant changes between the spectra of HT and NT samples. NT indicates normotensive Dahl/SS rat; HT, hypertensive Dahl/SS rat; LV, left ventricle; RV, right ventricle; LA, left atrium; and RA, right atrium.

In HS6 animals, the second derivative of the FTIR spectra of atria and ventricles revealed significant differences at 1654 cm^−1^, 1550 cm^−1^ and 1396 cm^−1^ for the RV and LA (Fig. 3a). Data indicated a greater amount of protein (amide bands), and free amino acids (1396 cm^−1^ signal)^12^ in the HT samples compared with the NTs. The 1396 cm^−1^ signal is assigned to a vibration of the carboxylic group of amino acids (CO_2_^−^ symmetric stretching), which is not specific to a single species but is common to all the amino acids.

Using Raman spectroscopy, we observed significant differences in the amide bands for the RV and, to a minor extent, for the LA (Fig. 3b). Specifically, the amide III band was more intense and the amide I band was blue-shifted in the spectrum of HT with respect to NT samples.

**Figure 4:**
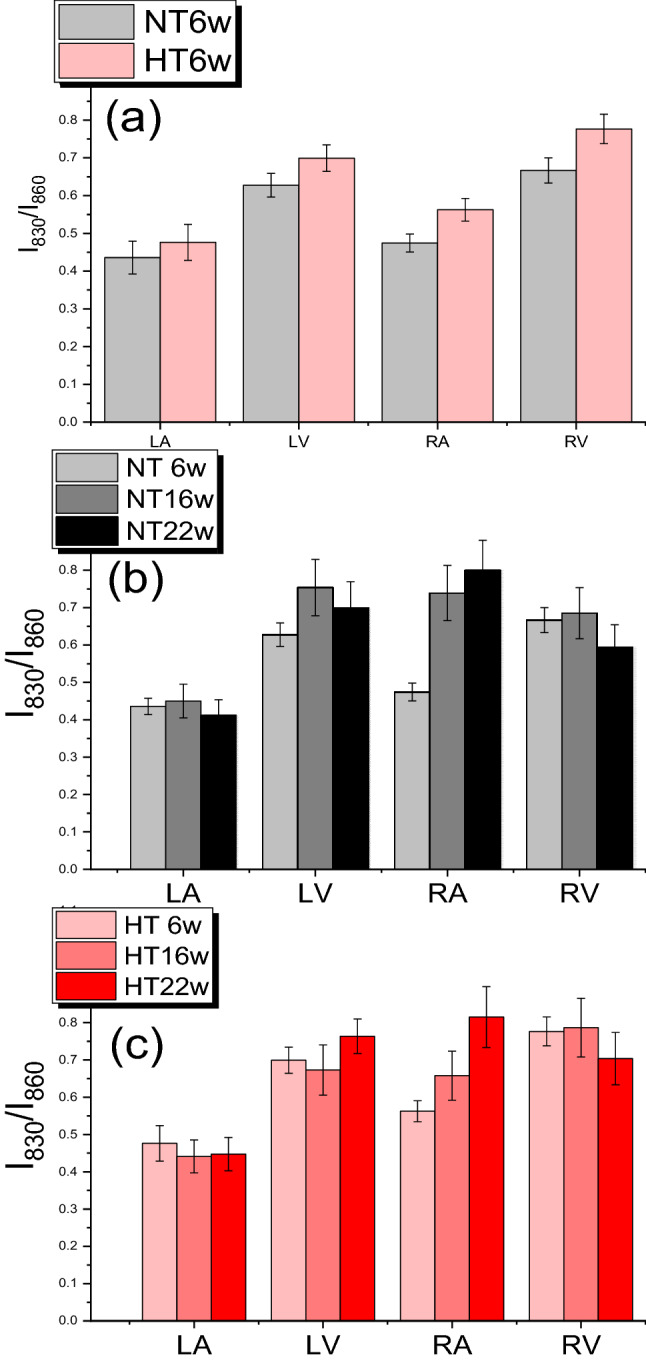
(**a**) 830/860 cm^−1^ intensity ratio (I_830_/I_860_) obtained from Raman spectra of six-week normotensive and hypertensive rats. I_830_/I_860_ obtained from Raman spectra of (**b**) normotensive and (**c**) hypertensive rats at six- (6w), 16- (16w), and 22-weeks (22w) of age. NT indicates normotensive Dahl/SS rat; HT, hypertensive Dahl/SS rat; LV, left ventricle; RV, right ventricle; LA, left atrium; and RA, right atrium.

Other significant differences were observed in the intensity of the band at 830 cm^−1^ (I_830_) when this was assessed using the 860 cm^−1^ intensity (I_860_) as a reference (the intensity of this band was constant from sample to sample). In tissues obtained from HS6 Dahl/SS rats, the I_830_/I_860_ ratio provided three types of information: (a) it was higher in ventricles compared with atria of control tissues; (b) it was higher in HT with respect to NT samples of the same chamber; and (c) for tissues of HT rats, its increase was higher in the RA and RV with respect to their left counterparts (Fig. 4a). The same parameter was also determined in heart samples of HS16 and HS22 Dahl/SS rats (Fig. 4b, c). Apart from the RA, little variation was observed due to an ageing effect.

**Figure 5:**
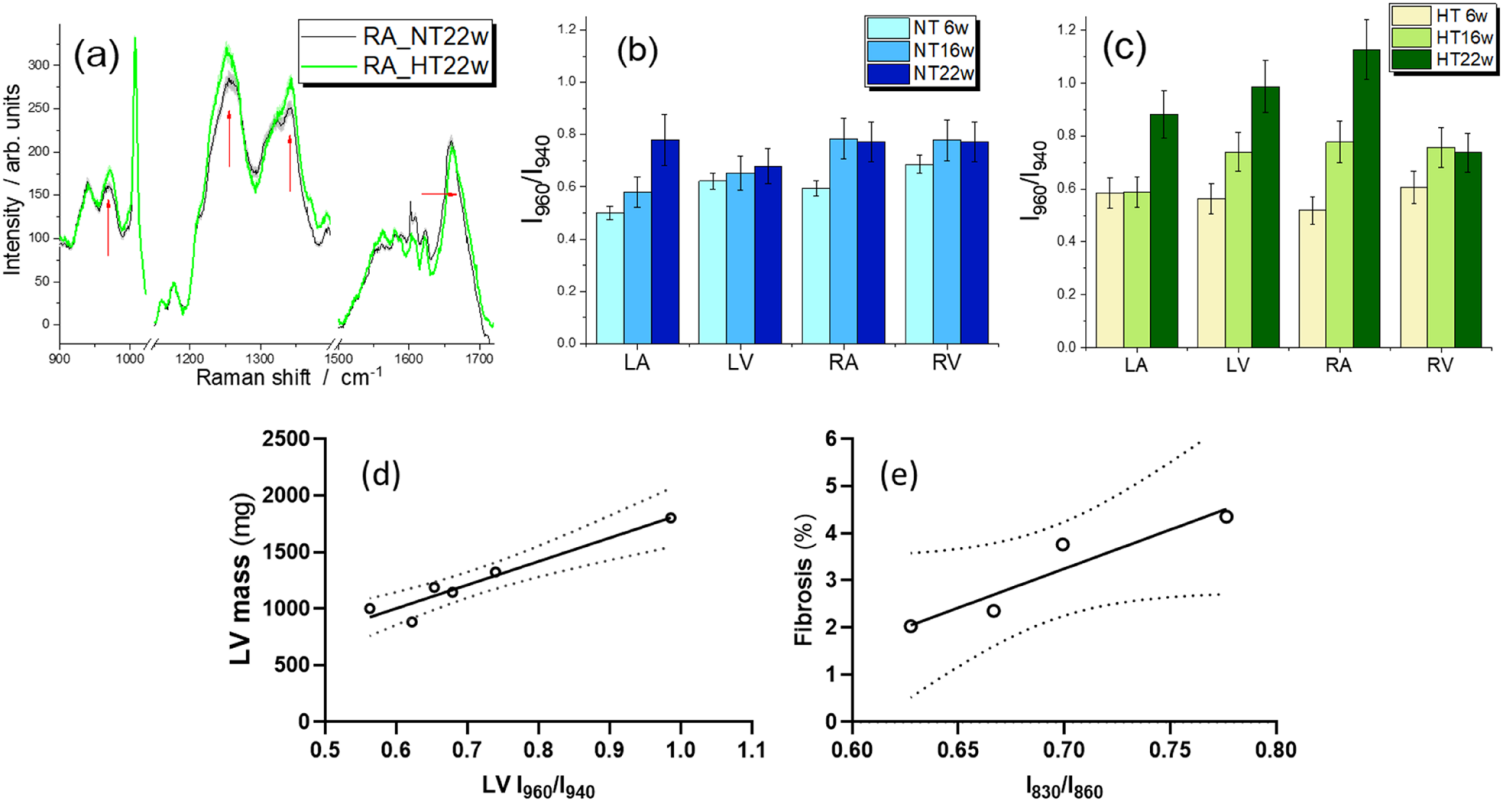
(**a**) Raman spectra of normotensive and hypertensive 22-week-old rats. Red arrows indicate significant changes between spectra of HT and NT samples. 960/940 cm^−1^ intensity ratio obtained from Raman spectra of (**b**) normotensive and (**c**) hypertensive rats at six- (6w), 16- (16w), and 22-week-old (22w) rats. Linear correlation between the LV I_960_/I_940_ ratio and LV mass, and ventricular I_830_/I_860_ ratio and interstitial fibrosis, with 95% confidence interval (dotted lines), is shown in (**d**) and (**e**), respectively. NT indicates normotensive Dahl/SS rat; HT, hypertensive Dahl/SS rat; LV, left ventricle; RV, right ventricle; LA, left atrium; and RA, right atrium.

A robust variation of amide III intensity was observed in cardiac samples from the HT Dahl/SS rats after 22 weeks, predominantly in the RA (Fig. 5a). In addition, we observed a significant increase of intensity at 960 cm^−1^ (I_960_) for the LA and RA of the control animals. This intensity progressed from 6 to 22 weeks of age (Fig. 5b). We evaluated the relative intensity of this feature with respect to the signal at 940 cm^−1^ (I_960_/I_940_); the latter is characteristic of both proteins and lipids and is referred to as an internal standard^13^. Higher I_960_/I_940_ values were estimated for tissues from HT rats (Fig. 5c) and the increase of this ratio from NT to HT was particularly notable in the LV and RA of the HS22 animals. A positive correlation (r^2^ = 0.9224, *p* = 0.0012) was observed between the I_960_/I_940_ ratio and LV mass (Fig. 5d; Table 2). A positive correlation (r^2^ = 0.8864, *p* = 0.0292) was also observed between levels of cardiac interstitial fibrosis and the I_830_/I_860_ ratio, as detected by Raman spectroscopy (Fig. 5e; Table 2).

**Table 2:** Pearson correlation coefficients (r^2^) and *p* values for the relation of Raman/FTIR spectral bands (I_830_/I_860_ and I_960_/I_940_) with cardiac functional and structural measurements.

Variables	I_830_/I_860_		I_960_/I_940_	
	r^2^	*p* value	r^2^	*p* value
**Echocardiogram**				
TV LW A′ (mm/s)	0.0005	0.4890	0.3972	0.0899
TV LW E′ (mm/s)	0.0008	0.4858	0.0857	0.2867
TV LW E′/A′	0.0060	0.4611	0.1685	0.2094
MV E/A	0.0015	0.4707	0.1612	0.2150
LV mass	0.4898	0.0608	0.9224	**0.0012**
LA dimension	0.2507	0.1558	0.0643	0.3139
**Histology**				
Interstitial fibrosis (%)	0.8864	**0.0292**	0.3864	0.1892
Perivascular fibrosis (%)	0.3057	0.2235	0.7516	0.0665

TV LW A′, peak velocity of diastolic mitral annular motion as determined by pulsed wave Doppler; TV LW E′, peak velocity of early diastolic mitral annular motion as determined by pulsed wave Doppler; TV LW E′/A′, ratio of E′ to A′; LV mass, left ventricle mass; MV E/A, mitral inflow velocity ratio of E to A; LA dimension, left atrium dimension.

## Discussion

The largest differences in the FTIR spectra of the four heart chambers of NT samples were seen in the amidic bands of proteins. The RA had the highest intensity of these amidic bands. These results are in agreement with those of Pelouch et al.^14^, who reported that the total protein content of the atria was greater than that of the ventricles, particularly in the right chamber. In Raman spectra of atria, an increase of intensity was observed at about 1250 cm^−1^ and the amide I band showed a blue shift. These two effects could be correlated with increasing collagen concentrations in the tissue^15,16^.

An amide I differentiation of atrial and ventricular Raman spectra was recently observed by Brauchle et al.^17^ for murine, paraffin-embedded samples. We confirmed this observation and provided evidence of other significant differences between the two types of cardiac chambers. Our results showed higher intensity of 830 cm^−1^ and 903 cm^−1^ Raman bands in LV and RV samples. The assignment of features in the 800–1000 cm^−1^ spectral region is difficult, since vibrations from various chemical species contribute. For instance, in the region between 850 and 940 cm^−1^, different C–C stretching modes of proline and hydroxyproline can be recognized^18^. Our observations indicated that the intensities of the 830 cm^−1^ and 903 cm^−1^ bands were strictly related, which suggested that they were due to tyrosine (tyr) vibrations^19^; it has been reported that tyr signals are always clearly visible in the Raman spectra of muscle cells^13,20^. We estimated the ratio of the intensity of the 830 cm^−1^ Raman band to that of the 860 cm^−1^ feature. The 830–860 cm^−1^ couple is known as the tyr-Fermi doublet. The I_830_/I_860_ ratio has traditionally been assumed to indicate the extent of hydrogen-bonding interactions on phenolic OH; the stronger the interaction with OH as a proton-donor group, the higher the ratio^19^. Therefore, the variation in the relative intensity that we observed (Fig. 2) could be explained by a different exposure of tyr residues to the solvent followed by a conformational change of tyrosine-rich proteins, or a different degree of phosphorylation or methylation of these residues^21^. Alternatively, due to the presence of proline/hydroxyproline signals in the same spectral range, it could be related instead to a different collagen content or to a different structure of collagen fibers^18^.

The Dahl/SS rat is an established model of renal and cardiovascular disease that is induced by high blood pressure when the rats are fed a HS diet^22^. Our experimental model mimics the early onset of HFpEF in its transient phase while biochemical changes in the heart are already present. Rats that were fed a HS diet exhibited a progressive increase in levels of NT-proBNP, which was, however, ascribable to ageing and not to the altered function or structure of the heart, as it did not differ from a similar increase reported in the age-matched, normal controls. The low sensitivity of this biomarker that was observed in our study is consistent with previous investigations in humans, which have shown a lack of activation of this hormone in the early phase of cardiac dysfunction and hypertension^23–25^. Thus, this humoral biomarker was not useful to detect such alterations. Moreover, while the prognostic value of NT-proBNP in HFpEF has been established, some controversy remains regarding its relevance as a diagnostic tool for this condition, due to the lower circulating levels of NT-proBNP compared with those that are found in HF with reduced ejection fraction.

Our intention was to determine cardiac biochemical fingerprints in a comprehensive medical window that ranged from the early stages of adverse cardiac remodeling to onset of HFpEF. Accordingly, we performed our analysis in Dahl/SS rats that showed signs of diastolic dysfunction after six weeks of HS diet. These signs were a moderate increase of blood pressure, increased thickness of the heart walls, enlarged LA, concentric remodeling and fibrosis. The signs of advanced HFpEF were seen in the HS22 rats and in HS16 Dahl/SS animals that showed transition from early diastolic dysfunction into HFpEF. These signs were hypertension, diastolic dysfunction and concentric hypertrophy. However, the chemical modifications described above (i.e. protein, free amino acids and tyr accumulation) were detectable at six weeks of HS diet, prior to the manifestation of canonical signs of adverse remodeling (hypertrophy, fibrosis, diastolic dysfunction, NT-proBNP elevation, etc.).

Parallel to the increases in hypertrophy and RWT, we observed robust increases of I_960_/I_940_ values in the spectra of LV and RA chambers of HT rats (Fig. 5). The 960 cm^−1^ Raman band was assigned to hydroxyapatite, the mineral phase that is found at the early stage of vascular calcification^26,27^. We observed a positive correlation between the LV I_960_/I_940_ ratio and LV mass, which suggested a relationship between increased mineral deposits and increased cardiac size. The physiochemical relationship between the two warrants further investigation, in particular of the cellular or extracellular localization of the deposits. Elevated levels of Ca^2+^ in the resting cytosol are a feature of HFpEF^28^, and vascular calcification is implicated in the development of HFpEF^29^. Our data suggest that this signal is an efficient marker of this pathological condition; therefore, it may serve as an early diagnostic tool if we consider the use of Raman spectroscopy in-vivo^30^.

Recent insights have revealed the prognostic importance of LA dysfunction in HFpEF^31,32^. While most attention has been on the remodeling of the LV, evidence suggests that events in the LA and RV contribute to HFpEF etiology. Indeed, LA dysfunction may explain the pulmonary congestion, shortness of breath, and exercise intolerance that are associated with HF. Accumulating evidence indicates that right HF is the leading cause of death in patients with HFpEF^8^. We realize the challenges that applying this method to the clinical setting poses. Our strategy is to combine these imaging techniques with conventional invasive catheterization approaches.

Our FTIR analysis indicated higher protein content in the RVs and LAs of HT rats compared with the controls. In addition, we also observed an intensity change at 1396 cm^−1^, which might be due to altered metabolism in the myocytes of HT rats that leads to an increase of free amino-acid concentration in general; other analytical techniques (i.e., HPLC or mass spectrometry) could probably help recognize if there is one particular species showing a higher concentration in tissues from hypertensive rats.

High protein and amino-acid intakes have been inversely associated with arterial stiffness and blood pressure^33^. Thus, our results could be related to altered metabolism that leads to the accumulation of polypeptidic species at the onset of the pathological cardiovascular condition. Interestingly, the Raman findings for the I_830_/I_860_ band ratio could be correlated significantly with the histological assessment of total collagen deposition in the cardiac tissue (for both RV and LV chambers). Besides, the amide III Raman band indicated increased collagen content in samples of the RV. This evidence suggests that both tyrosine and collagen bands are efficient indicators of interstitial fibrosis. One could think that a relationship exists between the increase of IR band at 1396 cm^−1^ and the increase of the I_830_/I_860_ Raman intensity ratio, but considering the different heart chambers and the stage of the pathology, the correlation is not observed. So, we conclude that the two techniques have a different sensitivity to monitor the effects of HFpEF.

In conclusion, this study has demonstrated the occurrence of early chemical changes (i.e. in collagen, tyrosine, protein and free amino-acid accumulation) during the evolution of HFpEF that precede the onset of canonical and often belated signs of cardiac remodeling and which are detectable with conventional diagnostic tools. Importantly, through use of FTIR and Raman spectroscopy, we are able to detect differently regulated chemical and structural changes among the four chambers of the heart and demonstrate unprecedented sensitivity and specificity. It is possible that the different pressures, and the consequent different stimulations, of the mechanosensors of the four chambers could have led to the different biochemical modifications that were observed in the atria and the ventricles. Several studies have underscored the role of mechanical force as a regulator of the structure and function of cells, tissues and organs^34^. The role of mechanosensors in the regulation of the biochemical changes is unclear and further studies are warranted. Future studies will need to be performed to address how specific the changes we observed are for HFpEF. It also remains to be evaluated whether early detection of chemical alterations can provide novel therapeutic targets for the treatment of HFpEF and whether early diagnosis can lead to improved prognoses in such patients.

## Supplementary Information

Supplementary Figure S1.

Supplementary Figure S2.

Supplementary Figure S3.

Supplementary Information 1.

## Data Availability

All protocols, data, and materials used to conduct the research will be made available to any researcher for purposes of reproducing the results or replicating the procedure.

## Abbreviations

Dahl/SS: Dahl/salt-sensitive
FTIR: Fourier-transformed infrared
HF: Heart failure
HFpEF: Heart failure with preserved ejection fraction
HS: High salt
HT: Hypertensive
NT: Normotensive
LA: Left atrium
RA: Right atrium
LV: Left ventricle
RV: Right ventricle
NT-proBNP: N-terminal pro b-type natriuretic peptide
SBP: Systolic blood pressure
DBP: Diastolic blood pressure
MAP: Mean arterial pressure
BW: Body weight
HW: Heart weight
EF: Ejection fraction
FS: Fractional shortening
E/A: Mitral valve E velocity over mitral valve A velocity
LV Vol;_d_ or ;_s_: Left ventricular volume in diastole or systole
IVS;_d_ or ;_s_: Interventricular septum thickness in diastole or systole
LVPW;_d_ or ;_s_: Left ventricular posterior wall thickness in diastole or systole
TV LW A′: Peak velocity of diastolic mitral annular motion as determined by pulsed wave Doppler
TV LW E′: Peak velocity of early diastolic mitral annular motion as determined by pulsed wave Doppler
TV LW E′/A′: Ratio of E′ to A′
MV E/A: Mitral inflow velocity ratio of E to A
FFPE: Formalin-fixed, paraffin-embedded
