# Supplementary figures and images for: Early cardiac-chamber-specific fingerprints in heart failure with preserved ejection fraction detected by FTIR and Raman spectroscopic techniques
Source: Sci Rep. 2022 Mar 2;12:3440. doi: 10.1038/s41598-022-07390-2 (PMC8891318; doi:10.1038/s41598-022-07390-2)

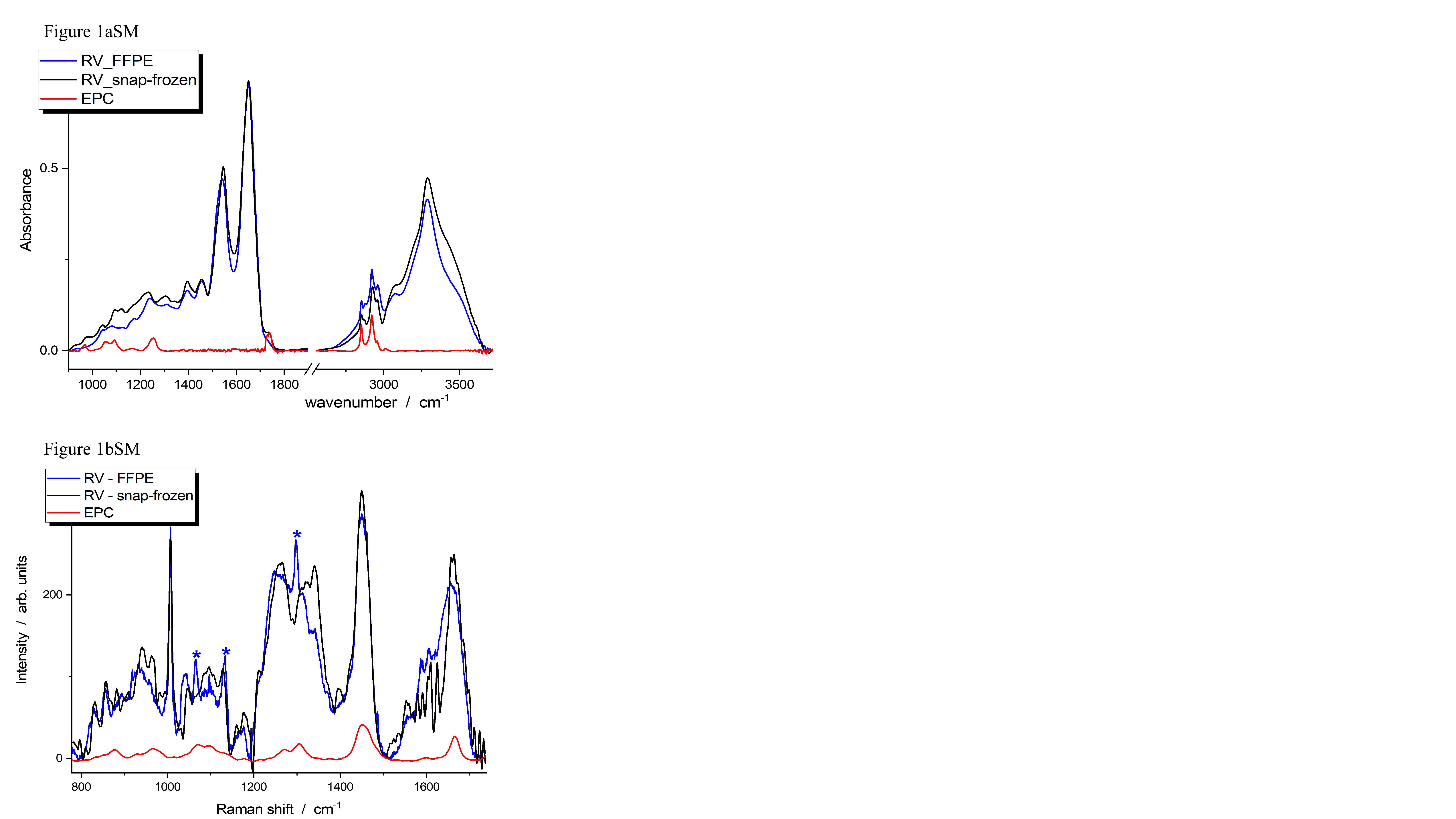

Supplement: Supplementary file 1 — Supplementary Figure S1. [file 41598_2022_7390_MOESM1_ESM.tif]

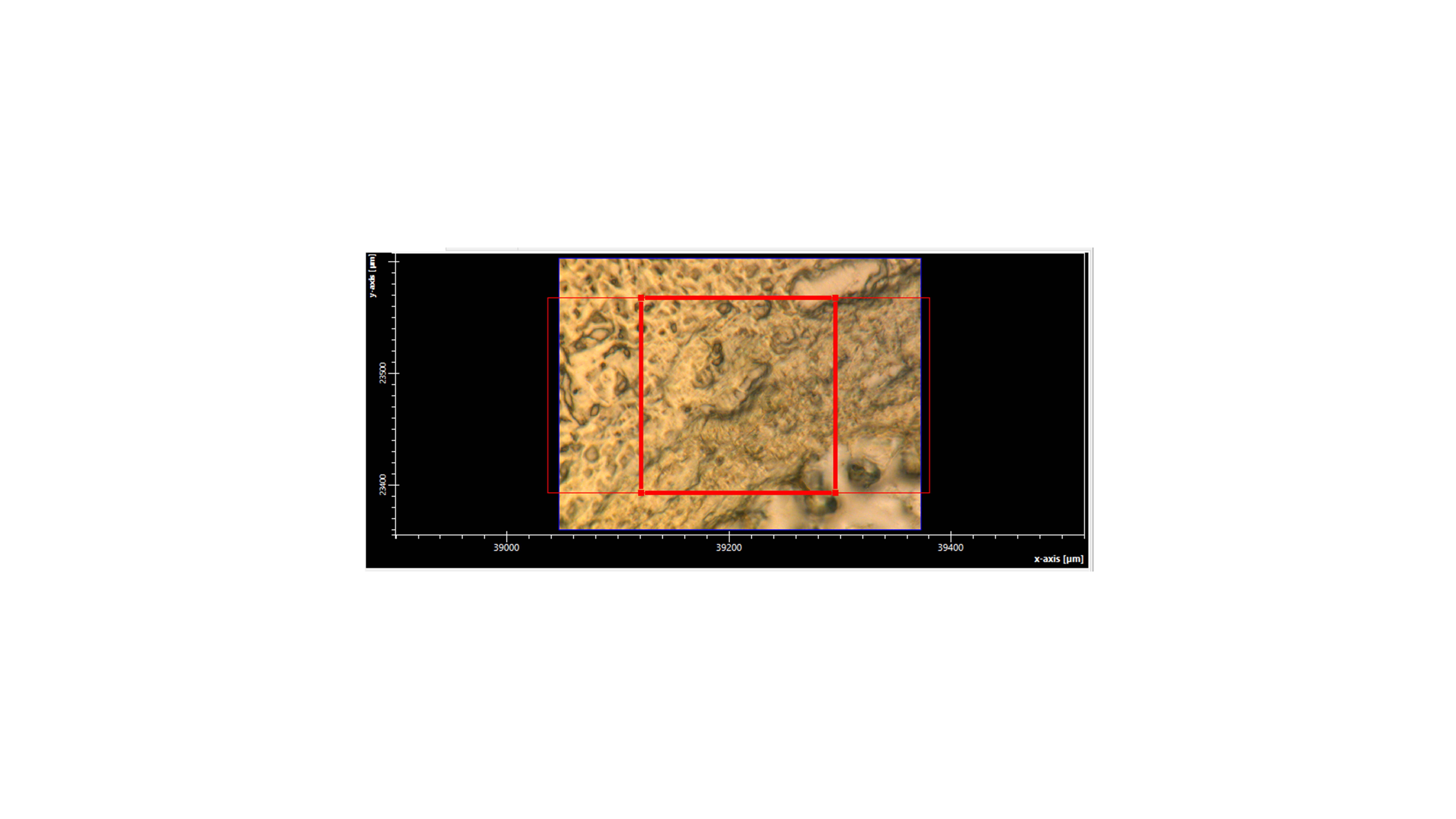

Supplement: Supplementary file 2 — Supplementary Figure S2. [file 41598_2022_7390_MOESM2_ESM.tif]

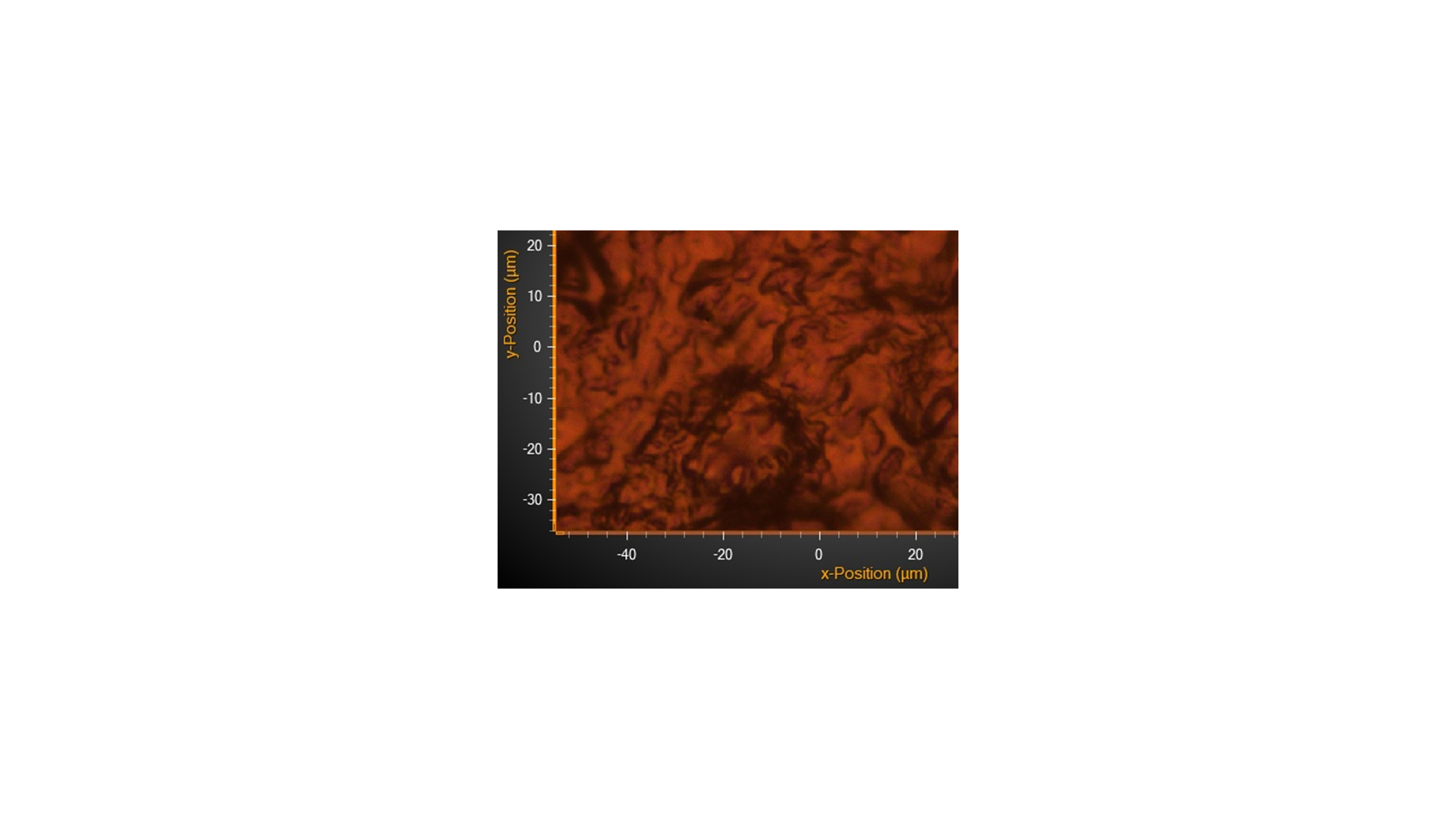

Supplement: Supplementary file 3 — Supplementary Figure S3. [file 41598_2022_7390_MOESM3_ESM.tif]
