# Supplementary material for: Early cardiac-chamber-specific fingerprints in heart failure with preserved ejection fraction detected by FTIR and Raman spectroscopic techniques
Source: Sci Rep. 2022 Mar 2;12:3440. doi: 10.1038/s41598-022-07390-2 (PMC8891318; doi:10.1038/s41598-022-07390-2)
Supplement: Supplementary file 4 — Supplementary Information 1. [file 41598_2022_7390_MOESM4_ESM.docx]

**Supplementary material**

**Sample preparation of** **formalin-fixed and paraffin-embedded samples**

The apical portion of the heart was formalin-fixed and paraffin-embedded (FFPE). Briefly, samples were submerged in 10% formaldehyde for 24h at room temperature, and then preserved in 70% EtOH until the paraffin inclusion. FFPE samples were sectioned using the paraffin wax process. They were dehydrated in multiple steps from 70% to absolute ethanol. Xylene was then used for the clearing stage prior to paraffin wax infiltration. Slices of thickness 5µm and 10µm were obtained from the embedded samples and deposited on BaF_2_ infrared (IR) discs for FTIR and Raman analyses. Paraffin was then removed completely through performance of a double wash in xylene. Finally, the samples were partially rehydrated in multiple steps from absolute to 70% alcohol. Snap-frozen samples were sliced by cryosection. Small pieces of tissue were sliced in a pure water embedding in order to avoid any contamination related to the use of an optimal cutting temperature compound. Sections 6µm thick were cut and deposited on BaF_2_ IR discs for FTIR analysis. For Raman analysis, an additional set of ~1mm-thick, manually sliced sections were deposited on an aluminum substrate. Both preparations were left to dry at 37°C overnight.

**Biochemical differences between FFPE and snap-frozen heart tissue**

We compared the average FTIR spectra of FFPE and snap-frozen representative samples of the heart tissue. In **Fig. 1aSM** can be seen the FTIR spectra of the right ventricle of FFPE and snap-frozen tissues of control animals. The comparison between spectral profiles shows how much the results are influenced by the sample treatment prior to spectra acquisition. Significant differences are particularly clear in the 1000-1300cm^-1^ and 1720cm^-1^ regions. At these wavenumbers, the characteristic bands of the phosphate and carbonyl groups of phospholipids can be observed. This is confirmed by the spectral profile of a model phospholipid, egg palmitoylphosphatidylcholine (EPC), which is shown in **Fig.1aSM**. The lower intensity of these bands in the spectrum of the FFPE tissue compared with that of the snap-frozen sample suggests a lower phospholipid concentration in the FFPE tissue than in the snap-frozen sample. However, the CH_2_ symmetric and asymmetric stretches that are shown at 2850cm^-1^ and 2920cm^-1^, which are mainly related to long saturated lipid chains, maintain the same ratio to the intensity of amide bands. This is evidence that some short-chain phosphatidic acid compounds were extracted during preparation of the FFPE sample.

The same samples that had been analyzed by FTIR were then assessed by Raman (**Fig.1bSM**). In a similar way to that which was observed with IR absorption, the Raman spectra of the FFPE tissues revealed a reduced intensity in the range of 800-1720cm^-1^, at wavenumbers where the characteristic peaks of lipids are normally observed. In addition, the intensities at about 1065, 1130 and 1300cm^-1^ indicated residual paraffin that altered the spectral profile. In our sample, both FTIR and Raman detection in the 800-1800cm^-1^ region revealed that some short-chain phosphatidic acid compounds were lost from the FFPE samples.

Figure 1aSM

Figure 1bSM

**Figure 1SM. (a)** FTIR and **(b)** Raman spectra of snap-frozen (black line) and FFPE (blue line) samples of the right ventricle. Differences are compatible with the signals of a model phospholipid-like egg phosphatidylcholine (EPC, red line). Marked differences between IR profiles are shown in the 1000-1300cm^-1^ and 1720cm^-1^ regions. In Raman analysis, the FFPE sample produces a spectrum with lower intensity signals at about 950, 1100, 1330 and 1660cm^-1^ than the snap-frozen sample. The FFPE samples show some characteristic paraffin bands (*) at 1065, 1130 and 1300cm^-1^. RV indicates right ventricle; FFPE, formalin-fixed paraffin-embedded; EPC, egg phosphatidylcholine.


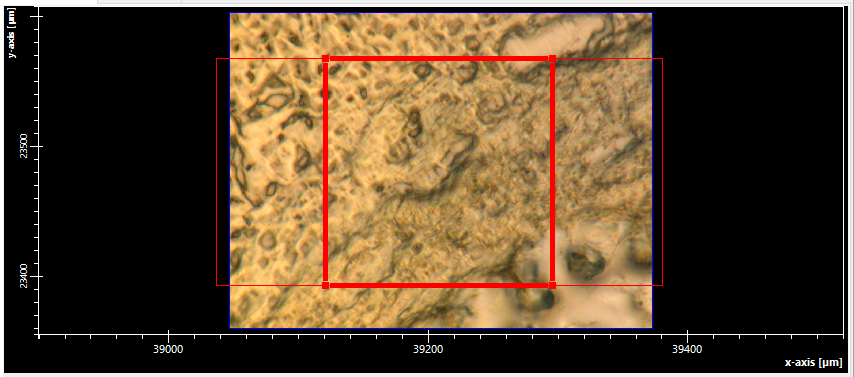


**Figure 2SM.** Optical micrograph (20X objective; N.A.= 0.4) of myocardial region of the right ventricle from a normotensive animal. The image was achieved by the Hyperion 3000 microscope; the area delimited by the red square identifies the measurement region for IR imaging.


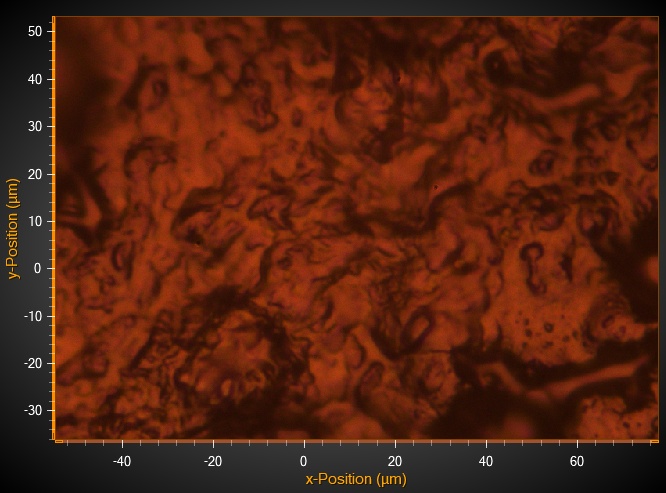


**Figure 3SM.** Optical micrograph (50X objective; N.A.= 0.5 ) of myocardial region of the right ventricle from a normotensive animal. Raman acquisition points (yellow circle) were selected and measured with the same objective in the flat regions of the visualized area.
